# Parental Concerns about Child and Adolescent Caffeinated Sugar-Sweetened Beverage Intake and Perceived Barriers to Reducing Consumption

**DOI:** 10.3390/nu12040885

**Published:** 2020-03-25

**Authors:** Allison C. Sylvetsky, Amanda J. Visek, Catherine Turvey, Sabrina Halberg, Jamie R. Weisenberg, Karina Lora, Jennifer Sacheck

**Affiliations:** 1 Department of Exercise and Nutrition Sciences, Milken Institute School of Public Health, The George Washington University, 950 New Hampshire Ave NW, Washington, DC 20052, USA; avisek@gwu.edu (A.J.V.); cturvey@gwmail.gwu.edu (C.T.); halbergs@gwmail.gwu.edu (S.H.); jrweisen@syr.edu (J.R.W.); klora@email.gwu.edu (K.L.); jsacheck25@email.gwu.edu (J.S.); 2 Sumner M. Redstone Global Center for Prevention and Wellness, Milken Institute School of Public Health, The George Washington University, 950 New Hampshire Ave NW, Washington, DC 20052, USA

**Keywords:** sugar, caffeine, childhood obesity, beverage consumption, soda

## Abstract

Sugar-sweetened beverage (SSB) consumption contributes to obesity and chronic disease. SSB intake in children and adolescents remains well above recommendations and reducing intake is challenging. In addition to high sugar content, SSBs are the predominant source of caffeine among youth. However, whether caffeine in SSBs presents unique barriers to reducing consumption is unknown. Herein, we examine parental concerns about child caffeinated-SSB (CSSB) intake and describe parent-reported barriers to lowering their child’s consumption. In-depth qualitative interviews were conducted with 21 parents of children and adolescents 8–17 years of age. Interviews were audio-recorded and transcribed verbatim. Transcripts were coded using Nvivo™, and key themes were identified. Most parents expressed concern about child CSSB consumption, primarily with regard to dietary (e.g., excess sugar), health (e.g., obesity, diabetes) and/or behavioral (e.g., hyperactivity) consequences of frequent intake. Several key barriers to CSSB restriction were reported, encompassing six emergent themes: widespread availability and accessibility; child non-compliance when asked not to drink CSSBs; peer and cultural influences; negative child response to CSSB restriction; family eating behaviors; and, child preferences for CSSBs versus other beverages. Consideration of these barriers, along with the development of novel approaches to address these challenges, will likely bolster success in interventions aimed at reducing CSSB intake among children and adolescents.

## 1. Introduction

Sugar-sweetened beverage (SSB) consumption is associated with increased risk of dental caries, as well as excessive weight gain and obesity among children [1,2]. Obesity increases the risk of bone and joint issues, sleep disturbance, and psychological problems during childhood, as well as the risk of cancer, type 2 diabetes, and heart disease later in life. Over 60% of children in the United States (U.S.) drink SSBs daily [3] and children of African American or Hispanic race or of lower socio-economic status tend to have higher intakes [1].

A variety of correlates of SSB consumption, including price, taste, parenting practices, nutritional knowledge [1,4,5], availability of SSBs at home, screen time, and fast-food consumption, have been reported [6]. Differences in perceived social norms surrounding SSB intake across race/ethnic groups have also been described [7], which may be in part due to targeted marketing of unhealthy foods and beverages to minority populations [8].

In addition to having high sugar content, SSBs are also the main source of caffeine among children [9]. Yet, most studies evaluating effects of SSBs on children focus on sugar content. However, the combination of sugar and caffeine in caffeinated sugar-sweetened beverages (CSSBs; e.g., colas and sweet teas) may be uniquely reinforcing [10]. Repeated caffeine intake causes dependence in adults [11] and compelling evidence for sugar dependence is emerging [12]. While a variety of factors, including palatability, accessibility, publicity, affordability, and social acceptability contribute to frequent and sustained SSB consumption [7,13], the combination of caffeine and sugar in CSSBs may further encourage continued intake [14]. Prior studies have focused on SSBs regardless of whether or not the SSBs are caffeinated [7,13,15,16]. It is therefore critical to address the paucity of knowledge surrounding CSSB consumption in children.

With regard to CSSBs, studies have reported that the primary driver of consumption, as reported by adolescents, is their perceived physiologic effects, including increased energy, improved athletic performance, and appetite suppression [10]. Moreover, findings in both adolescents and adults have identified withdrawal avoidance and reversal or related psychostimulant effects as primary reasons for continued use [10]. Doses as low as 100 mg of caffeine per day produce withdrawal symptoms (e.g., headache, jitteriness, lack of concentration) in adults [17], and it is likely that lower doses could lead to caffeine dependence in children [10]. Given that a single 12 ounce can of caffeinated soda (e.g., Pepsi™, Coca-Cola™, Mountain Dew™) or 16–20 ounce bottle of sweet tea (e.g., Snapple™, Arizona™) contains 35–55 mg caffeine, it is critical to assess barriers specific to lowering CSSB consumption among children, which may differ relative to non-caffeinated SSBs.

The purpose of this study was to examine the extent to which parents are concerned about their children’s CSSB intake and to describe key barriers faced by parents in lowering their children’s consumption. This qualitative study is the first step of a larger research project investigating CSSB consumption among children and adolescents. Data generated from parent interviews guided the implementation of an intervention to investigate the feasibility of CSSB restriction among children and adolescents, primarily from low-income and minority backgrounds.

## 2. Materials and Methods

Qualitative, in-depth interviews were conducted with parents and caregivers (*n* = 21, hereafter referred to as parents) of children and adolescents 8–17 years of age, between October 2018 and April 2019. Interviews were conducted in English (*n* = 14) or Spanish (*n* = 7). Parents were recruited from community organizations primarily serving low-income, minority populations throughout the greater Washington, D.C. metropolitan area and recruitment ended once saturation of the data was reached. Inclusion criteria were parents’ reporting that (1) they had a child between eight and 17 years of age; (2) their child consumed ≥ 12 ounces of CSSBs (e.g., Coca-Cola™, Pepsi™, Mountain Dew™, Arizona Iced Tea™, etc.) per day (excluding energy drinks such as Red Bull™, Monster™, etc.); and, (3) they spoke English or Spanish fluently. Exclusion criteria were parents’ report that (1) their child consumed regular, caffeine-containing coffee, hot tea, or energy drinks ≥ 1 time per week; or (2) their child had diabetes. Regular energy drink consumption was an exclusion criterion because energy drinks are specifically marketed and consumed by youth to boost energy [18] and their consumption therefore likely reflects a different behavior compared to consumption of caffeinated, sugar-containing, soft drinks such as colas and sweet teas. Furthermore, consumption of caffeinated soda is far more prevalent than energy drink or coffee consumption among U.S. youth [19].

Eligibility was assessed by a trained study team member, in-person (on site at community centers), via e-mail, or by phone. The study protocol (#180445) was reviewed and approved by the Institutional Review Board at The George Washington University and is available upon request. All participants provided written informed consent prior to beginning the study procedures. Eligible parents were scheduled for a single, in-person, one-on-one interview, with the exception of one participant who completed the interview by phone, due to hazardous weather conditions, and two participants who completed the interview together, in English. Interviews were conducted by one of two trained interviewers (A.C.S., C.T.) in either English (A.C.S., C.T.) or Spanish (C.T.) at a community location (community center, library, etc.) most convenient for the participant. Prior to beginning the interview, the interviewer explained the protocol and asked the participant to complete a brief demographic and anthropometric questionnaire. No information on child demographics or anthropometrics were collected.

In-depth, semi-structured interview guides were developed collaboratively by three study team members (A.C.S., A.J.V., J.S.) and focused on determinants of child CSSB consumption, context surrounding child CSSB consumption, how the child obtained CSSBs, and any prior efforts and/or reasons for restricting child CSSB intake. Parent interviews were audio-recorded and lasted approximately 30 minutes. Participants were compensated with a $50 gift card for their participation.

Descriptive statistics, including means and frequencies, as appropriate, were used to analyze participant characteristics. All interview audio-recordings were transcribed verbatim. Interviews conducted in Spanish were translated into English by a certified translator and checked by a native Spanish speaker (S.H.). Two coders (A.C.S., C.T.) independently coded a subset of transcripts (*n* = 10) using the NVivo Pro software package (version 12; QSR International, Inc.; Burlington, MA, USA). The coders then developed a shared codebook, adding additional codes as they emerged. Previously coded transcripts were reviewed (A.C.S., C.T., J.R.W.) to ensure that all content was coded using the final shared codebook. Codes were compared to reach agreement between coders. Disagreement between coders were discussed until consensus was reached. After completing coding, the two coders examined nodes from all transcripts. Both coders read all nodes within each code category (e.g., what promotes CSSB consumption; what undermines CSSB restriction, etc.) and discussed the codes in order to reconcile any discrepancies. The two coders independently identified preliminary themes and then met to discuss differences in theme identification and potential addition of new themes or subthemes. The themes and subthemes identified were then discussed with a third research team member (K.L.), after which key themes and subthemes were further organized and condensed, and representative quotations were selected.

## 3. Results

Twenty-one parents participated in the qualitative interviews, due to reaching saturation of the data after 21 participants were enrolled. Parent demographic and anthropometric characteristics are shown in Table 1. All except for one parent was female (95%). A third (*n* = 7) of the parents self-identified as non-Hispanic Black and two-thirds self-identified as Hispanic (*n* = 14). Approximately half of the sample reported educational attainment of high school or less (*n* = 10), and two parents had completed a Bachelor’s degree.

Three key themes related to parents’ concerns about their children’s CSSB consumption emerged from the parent interviews (Table 2). A minor emergent theme was that some parents were not concerned about their children’s CSSB intake (Table 3) and did not believe it was important to lower consumption. Among the majority of parents who did express concern about their child’s CSSB intake, six themes captured key barriers to restricting consumption (Table 4).

### 3.1. Parent Concern about Child CSSB Consumption

#### 3.1.1. Concern about Health Consequences

A major theme was that parents were concerned about adverse health effects resulting from excess CSSB consumption. Parents frequently reported their child had gained weight, which prompted them to attempt to restrict CSSB intake. Concern about diabetes risk was also commonly reported, as parents often described experience with other family members developing diabetes and related complications. Furthermore, physician advice to limit CSSBs alerted parents to the adverse health consequences associated with CSSB intake, which in some cases prompted heightened parent efforts to restrict their child’s consumption.

#### 3.1.2. Concern about Child Diet

Insufficient water intake as a result of the child’s frequent CSSB intake was also commonly mentioned, particularly with regard to the color, smell, or volume of the child’s urine. Parents reported restricting CSSB intake until the child consumed a sufficient amount of water (as determined by the parent). High sugar intake resulting from CSSB intake was also alarming to parents, especially with respect to concomitant consumption of candy or junk food. Most parents recognized the unfavorable health effects associated with excess added sugar consumption.

#### 3.1.3. Concern about Behavioral Consequences

Parents expressed concern about changes in child behavior and/or sleep as a result of CSSB consumption and that drinking CSSBs made their child hyperactive, overly talkative, or loud. Similarly, CSSB consumption exacerbated child distractibility and impaired concentration in school. CSSB consumption, particularly in the evening, disturbed child sleep patterns and resulted in insufficient sleep. In some cases, parents described a cycle of continued CSSB intake as a means of staying awake or garnering energy after a night of poor sleep.

### 3.2. Lack of Parent Concern about Child CSSB Consumption

A minority of parents reported they were not concerned about their child’s CSSB intake. This was largely due to misconceptions about diet and health, particularly the belief that CSSBs have less sugar relative to other sweetened beverages. The misperception that excess sugar consumption was not unfavorable to health, as long as the child was not overweight, was also commonly described.

### 3.3. Barriers to Restricting CSSB Consumption

#### 3.3.1. Availability and Accessibility of CSSBs

Parents described that CSSBs were widely available and highly accessible, both geographically and financially. CSSBs were commonly obtainable in the home. Furthermore, the relatively low cost of CSSBs and the presence of retail outlets near the child’s home or school made CSSBs particularly obtainable. Parents reported that their child frequently stopped at nearby stores to purchase CSSBs on the way to or from school, often when unsupervised.

#### 3.3.2. Child Disobedience or Deception When Asked Not to Drink CSSBs

Children continued to obtain and consume CSSBs, even when instructed not to consume them. Parents reported children sneaking CSSBs, often by hiding beverages in their bedroom or closet, or by disguising them in water bottles or opaque containers. Some parents attempted to restrict their child’s CSSB intake by keeping CSSBs in a separate refrigerator in the parents’ bedroom, but these attempts were often defeated by children sneaking into the parents’ room without asking permission. Furthermore, some children concocted their own CSSBs (e.g., adding sugar and tea bags to water) when CSSBs were restricted.

#### 3.3.3. Peer and Cultural Influences 

Another emergent theme was that CSSB consumption was perceived as socially and culturally normative. Parents reported that they themselves and their child consumed CSSBs as a way of “fitting in” with family and friends. House guests and visitors expected that CSSBs be available, and thus, parents purchased CSSBs as a household staple. These beverages were also regarded by some parents as central to social gatherings.

#### 3.3.4. Negative Child Response to CSSB Restriction 

Parents reported that their child became angry and irritable if CSSBs were not provided. For example, a parent reported that their child would be rude and refuse to do household chores until a CSSB was provided. In some cases, children experienced physical symptoms, such as headaches or stomachaches when they did not drink CSSBs. These adverse reactions made it difficult to successfully restrict consumption, and in some cases, children were difficult to manage if requests for CSSBs were declined.

#### 3.3.5. Family Eating Behaviors

Family eating behaviors emerged as another main barrier to CSSB restriction. Parent modeling was pervasive, and many parents expressed guilt about normalizing CSSB consumption or introducing CSSBs to the child at a young age. Older siblings also model CSSB consumption and provide CSSBs to children who may not otherwise be able to obtain them. CSSBs were also perceived as a fundamental part of the family meal, particularly when eating at restaurants.

#### 3.3.6. Child Preferences for CSSB

Child preferences for CSSBs versus other beverages were described as a key barrier to effectively limiting consumption. Parents reported that their child enjoyed the taste of CSSBs, especially relative to water, and begged for CSSBs when they were restricted. Beyond their sweetness, sugar content, and palatability, children associated CSSB consumption with having more energy, which further reinforced consumption.

## 4. Discussion

Our findings demonstrate that most parents recognize frequent CSSB consumption as detrimental to their child’s health. Parents were aware that daily CSSB consumption increases risk of child weight gain and diabetes, as well as negative behavioral consequences such as hyperactivity and poor sleep. However, parents reported considerable difficulty in lowering their child’s CSSB intake, as a result of several reported barriers. These included widespread availability and accessibility of CSSBs, lack of child cooperation, peer and cultural influences, negative child responses to CSSB restriction, family eating behaviors, and child preferences for CSSBs over water or other unsweetened beverages.

Parental awareness of negative health effects associated with excessive sugar intake has been documented previously [20], specifically with regard to weight gain [21]. Concern regarding child hyperactivity and sleep problems resulting from SSB intake (not specific to CSSBs) have also been previously reported [20]. Several parents in the present study reported becoming concerned about CSSB intake after receiving counseling from their child’s pediatrician. This highlights an important, and often overlooked, role of pediatricians in dietary modification and childhood obesity prevention [22]. Park et al. [23] reported that only 23% of parents in a national survey recalled receiving counseling to modify child beverage intake from their child’s doctor, suggesting that strengthening counseling in pediatric primary care may be an effective means of encouraging parents to address their child’s beverage habits. However, all parents in our study had a child who consumed CSSBs daily (per inclusion criteria), despite expressing concern about CSSB-associated health effects. This paradox reiterates that simply educating parents about consequences of frequent CSSB intake is likely insufficient for lowering consumption [21].

Another interesting finding was that a small subset of parents viewed CSSB restriction as unnecessary, and perceived CSSBs to be harmless unless the child was overweight. This is especially concerning given that parents often do not recognize that their children are overweight or have obesity [24]. Furthermore, parents’ lack of general nutrition and health knowledge sometimes counteracted well-intentioned efforts. For example, several parents reported they allowed their child to consume sweet tea instead of soda, due to their perception that it is lower in sugar. These misconceptions suggest that nutrition education surrounding what constitutes a healthful beverage and clarification regarding adverse consequences of CSSB intake, beyond weight gain, may be warranted.

Although the majority of parents expressed desire to reduce their child’s consumption, availability of CSSBs in the home was repeatedly cited as a key barrier, consistent with prior reports [5,13]. In several cases, CSSBs were available because parents or other family members consumed these beverages. This finding is supported by a large body of research underscoring the importance of caregivers’ modeling in influencing child dietary behaviors [20,25]. Most parents acknowledged the contribution of their own CSSB consumption to their child’s intake, and several reported feeling guilt for introducing CSSBs and/or making them available in the home. These findings highlight the need to design interventions that target the whole family [26], rather than focusing solely on modifying child intake, and suggest that replacement of CSSBs with healthier alternatives, such as water, may be particularly critical in the context of the home environment.

Some parents attributed having CSSBs available in the home to social and/or cultural expectations. CSSBs were purchased as a household staple to ensure that they were available for visitors. Other parents reported that their child viewed CSSB consumption as a way to fit in with their friends. The importance of normative beliefs in influencing dietary behavior is well described [27], and interventions aimed at altering cultural and social norms surrounding CSSBs may therefore be helpful in discouraging intake. Importantly, modification of social norms has been successful in reducing other risk behaviors, such as cigarette smoking [28], and the key impact of peer influences on youth behavior is well documented [29]. Changing peer perceptions of CSSB consumption, perhaps through engagement of social influencers, may hold particular promise in lowering consumption among children [30] and modifying parental CSSB-related perceptions and behaviors.

Other commonly described barriers to lowering CSSB intake included proximity to retail outlets such as convenience stores, gas stations, and fast-food restaurants. Higher SSB intake among adolescents who attend school near a fast-food outlet has been reported previously [31], and elevated CSSB consumption when eating out, particularly at fast-food venues, is well documented [32,33]. Parents frequently mentioned their child purchased CSSBs going to or from school, often with the help of older siblings. Children in urban settings (e.g., Washington, D.C.) often walk or take public transit to or from school or activities (e.g., after school, sports, daycare, etc.), in most cases without parent supervision. Thus, this time period represents an important potential target for future efforts to reduce SSB consumption. This finding also reiterates the importance of addressing the neighborhood food environment, particularly in urban settings and low-income communities, where access to fast food and junk food is often high relative to healthier options [34,35].

An unsurprising barrier to CSSB restriction was that their child preferred them over other beverages, such as water. Parents reported that children were displeased, angry, aggressive, and disobedient when CSSBs were restricted. Restricting CSSBs frequently led to the child begging for CSSBs, and some parents felt it was easier to give in to their child’s demands. While child liking of sugar and preference for sweetness are well established [36], parents also reported that children consumed CSSBs to boost their energy. In fact, some parents described a cycle where the child drank CSSBs to combat fatigue, but consumption then interfered with the child’s sleep, resulting in further fatigue and continued CSSB intake.

The idea that children may depend on CSSBs to stay awake parallels well-established behavioral patterns surrounding coffee consumption in adults [37]. Particularly noteworthy were reports that some children developed symptoms consistent with withdrawal, such as onset of headaches, depressed mood, and social isolation, when CSSBs are restricted. This highlights a need to investigate whether children may be physiologically or psychologically dependent on these beverages, which contain caffeine along with large quantities of added sugar.

### Strengths and Limitations

A key strength of our study was recruitment of a sample of Hispanic and non-Hispanic Black parents throughout the Washington, D.C. metropolitan area. While this study was not designed to detect racial and ethnic differences, the findings lay the groundwork to develop culturally specific strategies to lower CSSB consumption and promote more healthful beverage intake. Furthermore, this qualitative study is the first, to our knowledge, to specifically address parental views surrounding child CSSBs intake, as opposed to SSB consumption in general. The focus specifically on CSSBs calls attention to the possibility that combined consumption of caffeine and sugar may present unique barriers to restricting CSSB intake among children.

This study was also subject to several limitations, including the broad child age range for parental inclusion and our relatively small convenience sample of primarily mothers, which limits the generalizability of our findings. Importantly, our sample size was determined by reaching saturation, which is considered the leading indicator of sample size in qualitative studies [38]. Parent views reported in our sample also may not be representative of all parents of children who consume CSSB. Although we intentionally restricted the scope of the study to investigate CSSBs in order to specifically examine the unique combination of sugar and caffeine in CSSBs, it was difficult to determine what findings were unique to CSSB versus those that apply to SSB consumption in general. In addition, while both interviewers were trained and used an identical semi-structured guide to conduct the interviews, the use of two different interviewers may have biased the data generated. Finally, information on child weight status was not collected, and as with any study involving parent-report, results may have been influenced by social desirability bias.

## 5. Conclusions

Adverse health effects resulting from excess SSB consumption are well established [39]. However, public health efforts to lower intake have been met with limited success [40]. Our findings demonstrate that most parents are indeed concerned about their child’s CSSB intake but encounter significant barriers in effectively restricting their child’s consumption. These results reiterate that simply educating parents about detrimental health effects of CSSB intake is not sufficient to elicit behavior change. Parent-reported barriers could inform the design of future interventions, which may incorporate novel strategies to address negative child responses (e.g., withdrawal-like symptoms, negative affect, etc.) to CSSB restriction. These findings also highlight the need for comprehensive approaches focused on altering family eating practices and modifying cultural norms related to CSSB intake.

## Figures and Tables

**Table 1:** Characteristics of the Study Participants (*n* = 21).

N		21
Female (n (%))		20 (95%)
Race/Ethnicity (n (%))		
	Hispanic	14 (66%)
	Non-Hispanic Black	7 (33%)
Education ^1^ (n (%))		
	High school or less	10 (53%)
	Some college	7 (37%)
	Bachelor’s degree	2 (10%)
BMI ^2^ (mean ± SE)		33.0 ± 2.2
BMI ^1^ (range)		21.1–44.5

^1^ Education missing for two subjects; *n* = 19; ^2^ BMI missing for four subjects; *n* = 17.

**Table 2:** Parents’ Concerns about Child Caffeinated Sugar-Sweetened Beverage (CSSB) Intake.

Theme Subtheme	Select Representative Quotations (ID^1^)
**Theme 1: Concern about health consequences**	
Weight gain	*“We noticed that he was gaining weight, so we slowed it down little by little.” (ID 9)*
	*“They gained weight. They’re drinking all this stuff and they’re not as active as they used to be.” (ID 7)*
Diabetes	*“I tell her that my mom has diabetes and I say, ‘You want to be like your Grandmother pricking her fingers?’” (ID 14)*
	*“Somebody has to break the cycle. At the end of the day, there are a lot of people having diabetes, a lot of people who have kidney failure.” (ID 3)*
Pediatrician advice	*“We cut it out because we talked to the pediatrician, and she told me she’s very high weight. She told me I need to cut it out.” (ID 14)*
	*“After we went to the doctor, she said we shouldn’t have them, and then, I was making the conscious effort.” (ID 1)*
**Theme 2: Concern about child diet**	
Low water consumption	*“My twelve-year old’s pee—it’s strong. She needs to drink water. I tell her, ‘You can’t have any more sweet tea until you drink at least four bottles of water.’” (ID 16)*
	*“I notice when they use the restroom. I’m like this is just too yellow. It’s all orangey looking and it shouldn’t be like that and I know they need to drink more water.” (ID 1)*
Excessive sugar consumption	*“I don’t know if it’s the Mountain Dew™ sending little messages: “Don’t forget Starburst™.” I’m curious because I find that they [Mountain Dew™ and candy] always seem to go hand in hand.” (ID 15)*
	*“Even if there’s not caffeine, there’s just too much sugar in the drinks.” (ID 3)*
**Theme 3: Concern about behavioral consequences**	
Child lack of focus and/or hyperactivity	*“You’re drinking all of this sugar; it has you hype for a few minutes and then you just shut down. So, you’re drinking it to stay up in school, but at a certain point of time you’re still going to shut down.” (ID 7)*
	*“I’ve noticed that the more they drink, the more hyperactive they get.” (ID 20)*
Poor sleep	*“They would go bed very late and were getting up early to go to school. They weren’t sleeping the eight hours that they need. Because of this I tried to take it [soda] away.” (ID 12)*
	*I notice his sleeping pattern is off. He’s up, he cannot fall asleep, jittery, walking back and forth. He’s not going to drink soda anymore at night time.” (ID 4)*

**Table 3:** Parents’ Lack of Concern about Child Caffeinated Sugar-Sweetened Beverage (CSSB) Intake.

Theme Subtheme	Select Representative Quotations
**Theme: Lack of concern about child CSSB consumption**	
Healthier than other alternatives	*“It’s difficult to say; I’ll take them [CSSBs] away, but then the other option could be a lot worse. At the end of the day, it [the replacement] had a lot more sugar than the other drink” (ID 15)*
	*“I prefer [to give the child] sweet tea because sweet tea has less sugar than Coke™. The Coke™ is stronger than the sweet tea.” (ID 14)*
CSSB consumption not unhealthy	*“I haven’t seen a study out that says if you drink this amount of soda, you’re going to end up with cancer. I don’t think I have seen any studies that connect drinking soda to other health problems. Soda itself, I don’t see that it causes long-term health issues.” (ID 22)*
	*“He’s very adept with his life and everything that’s going on, so if it was [CSSB consumption] causing him some form of physical or mental anguish, he’d stop it [CSSB consumption].” (ID 15)*
Not problematic unless child is overweight	*“Sometimes you leave the kid alone, they’re growing, and they’re maturing. They’ll figure it out if they don’t have any pre-determined obesity or overweight or something like that.” (ID 15)*
	*“No one’s really overweight. So yeah, I think because they’re not overweight, it’s not a concern.” (ID 16)*

^1^ ID is used to attribute quotations to specific participants.

**Table 4:** Barriers to Restricting Caffeinated Sugar-Sweetened Beverage (CSSB) Consumption among Children.

Theme Subtheme	Select Representative Quotations
**Theme 1: Availability and accessibility of CSSBs**	
Home availability	*“It’s in the closet or the pantry. I don’t have a way to limit it.” (ID 17)*
	*“I’m going to say it’s my fault because I have Pepsi™ stacked. They’re so used to having soda in the house. I think that’s why my son turned to sodas.” (ID 4)*
Neighborhood food environment	*“We have a 7-Eleven that is right in walking distance and they’ll walk to 7-Eleven and get some Coke™, Pepsi™, orange soda, or whatever they feel like getting.” (ID 7)*
	*“She drinks at least one [CSSB] at school because I’m not there. There is a 7-Eleven across the street and she always gets one every day, either iced tea or Mountain Dew™.” (ID 14)*
Affordability	*“You know kids; when they have a dollar, instead of buying a bottle of water they go and get the Arizona™. It’s 99 cents. Most of the kids, especially the teenagers, buy the chips and Arizona™.” (ID 16)*
	*“It is easy for them to get them [CSSBs]. For example, their school is more or less a block from stores or a shopping center, and it is very cheap to buy these drinks.” (ID 12)*
**Theme 2: Child disobedience or deception when asked not to drink CSSBs**	
Obtain CSSBs despite parent efforts	*“We even hide the sodas at home, we hide them [in my room], but like I said, there’s always something [a CSSB] that she finds.” (ID 16)*
	*“It was time to clean their room. We found all these cups and two bottles of soda in there. I asked them, ‘Why would you go buy the whole bottle of soda’ and they were like ‘Because we wanted soda and we knew that you guys [parents] were not going to buy it.’” (ID 7)*
Sneaking in CSSBs	“*If I tell him no, he’s going to sneak the Pepsi™ in his room. I went to get the dirty laundry and I found a Coke™ right beside his bed.” (ID 4)*
	*“I ask him, ‘What are you bringing?’ He tells me, ‘water,’ but no, it’s soda.” (ID 20)*
**Theme 3: Peer and cultural influences**	
Influence of child’s friends	*“She went to Chick-fil-A and I guess someone said to order the sweet tea. She ordered it and ever since then it’s been like, ‘Oh I want iced tea, I want iced tea.’” (ID 2)*
	*“If her other friends are doing a Coke™ or Pepsi™, it’s like I am going to do that, kind of like peer pressure, like I need to fit in, I need to do this.” (ID 8)*
Influence of family friends and house guests	*“They [friends and family] bring Coke™. They all want Coke™. Then, in the house I always have to have soda because the people that come to visit me or my sister or my aunt they all want Coke™.” (ID 17)*
	*“Normally my friends always have soda. They’re like we know that when we go to her house, we have to bring something to drink because the only thing she has is water.” (ID 22)*
Cultural food norm	*“The majority of Latina people drink a lot of soda. So, since a young age we give it to our children.”* *(ID 18)*
	*“It’s a tradition in our household.” (ID 4)*
**Theme 4: Negative child response to CSSB restriction**	
Child’s anger	*“I’ll be like okay you don’t need this soda, you could drink water, and it’s like they get angry and they start getting aggressive.” (ID 7)*
	*“If I’m not going to give them soda they get mad…like he gets angry.” (ID 17)*
Symptoms consistent with withdrawal	*“He’ll get a little antsy, a little moody, talking at a fast pace. He will get very quiet and sometimes isolate himself.” (ID 4)*
	*“He gets headaches when he doesn’t drink it, and when he goes without drinking it for a day or two. It’s something heavy and his stomach hurts, but when he starts to drink [the soda], it doesn’t hurt anymore.”*
**Theme 5: Family eating behaviors**	
Parent modeling	*“My husband gets mad at me a lot because he says that I set the example for them [the children], and he is right.”(ID 10)*
	*“They’re [the children] the same way. It’s our fault. I teach the kids it’s normal to drink sodas.” (ID 14)*
Sibling influence	*“My 12 year old doesn’t get out of school until 4 o’clock, so the 16 year old goes to the store, gets her junk food, and then goes and get the twelve year old. And then they meet me at my job.” (ID 6)*
	*“When he arrives at school, his brother brings two sodas, because usually when he leaves work, he stops and buys soda. Both of them drink it.” (ID 10)*
Part of meals	*“They never eat without anything to drink. Food without a drink. There’s always something.” (ID 13)*
	*“It’ll be with lunch or with snack or with dinner or all three [meals].” (ID 15)*
Eating out	*“I believe that it’s a bad habit of the entire family because I recognize it. If I go to eat something good, out of obligation I feel like I have to get soda.” (ID 10)*
	*“Well every time we, for example, go to McDonalds or Pizza Hut or any restaurant he always asks for that. I mean, that’s his favorite drink.” (ID 9)*
Exposure at young age	*“It could be that as a baby he drank soda, who knows. I know that it’s really bad.” (ID 10)*
	*“Since they were little they’ve been drinking Coca-Cola™ because their mom loves Coca-Cola™. So, they’ve always been drinking Coke™ from since they were in elementary school.” (ID 7)*
**Theme 6: Child preferences for CSSBs**	
Taste	*“I believe more than anything that it’s because how rich in sugar it is and each time they buy, they want soda.” (ID 17)*
	*“Well, kids love sugars, you know, sweet drinks and all this stuff.” (ID 9)*
CSSBs are preferred over water	*“They would prefer to drink something sweet than to drink water. It doesn’t matter what it is, as long as it’s sweet, they’ll drink it, because they don’t want to drink water.” (ID 7)*
	*“She says it don’t taste like nothing.” (ID 16)*
Begging for CSSBs	“*It was hard because she’d ask, “Can I drink, can I drink, can I drink?” (ID 14)*
	*“He would be very insistent. ‘Mommy I want to go, Mommy I want to go buy [CSSBs], give me money.’” (ID 12)*
Perceived energy	*“They say it gives us energy. I’ve noticed that like they’re so tired and one of them said if we buy a soda we won’t be.” (ID 22)*
	*“I hear her conversation sometimes with other kids, ‘Soda’s good, it makes you stronger and have more energy.’ She relates that to she got a lot of energy when she got soda.” (ID 14)*
